# Do Statins Affect Cognitive Health? A Narrative Review and Critical Analysis of the Evidence

**DOI:** 10.1007/s11883-024-01255-x

**Published:** 2024-11-09

**Authors:** Richard Kazibwe, Rishi Rikhi, Saeid Mirzai, Nicklaus P. Ashburn, Christopher L. Schaich, Michael Shapiro

**Affiliations:** 1 Department of Internal Medicine, Wake Forest University School of Medicine, Winston-Salem, NC USA; 2 Department of Internal Medicine, Section on Cardiovascular Medicine, Wake Forest University School of Medicine, Winston-Salem, NC USA; 3 Hypertension and Vascular Research Center, Wake Forest University School of Medicine, Winston-Salem, NC USA; 4 Department of Emergency Medicine, Wake Forest University School of Medicine, Winston-Salem, NC USA; 5 Center for Preventive Cardiology, Wake Forest University School of Medicine, Winston-Salem, NC USA

**Keywords:** Statin, Cognition, Dementia, Cognitive impairment, Hypercholesterolemia

## Abstract

**Purpose of Review:**

Statins are the first-line treatment for hypercholesterolemia and play a key role in the prevention of cardiovascular disease (CVD). Current studies report mixed effects of statins on cognitive health, including harmful, neutral, and protective outcomes. However, these ongoing controversies about the potential cognitive adverse effects of statins may compromise their use in CVD prevention. Several factors may influence how statins affect cognition, including the unique cholesterol homeostasis in the brain, the limited permeability of the blood-brain barrier to lipoproteins, and the varying lipophilicity of different statins. This review examines the evidence linking statins to cognitive function and considers the effect of different dosages and treatment durations.

**Recent Findings:**

Earlier studies suggested cognitive disturbances with statins, but recent evidence does not strongly support a link between statins and cognitive impairment. In fact, observational studies suggest potential neuroprotective benefits, though biases like selection bias, confounding and reverse causation limit definitive conclusions. Two large randomized controlled trials, STAREE and PREVENTABLE, are underway, and their results are expected to address some of these gaps in the literature.

**Summary:**

Due to insufficient evidence in the current literature, well-designed randomized controlled trials are needed for a better understanding of statins’ effects on cognition. More data is needed regarding statin type, dose intensity, and treatment duration, which may affect cognitive outcomes. Future studies are also needed to examine how statins may affect cognition in specific high-risk groups, such as individuals with mild cognitive impairment, diabetes, cardiovascular disease, or chronic kidney disease.

## Introduction

Statins, also known as 3-hydroxy-3-methylglutaryl-coenzyme A reductase (HMGCR) inhibitors, are fundamental in the primary and secondary prevention of cardiovascular (CVD) [1]. Statins are among the most prescribed medications, with nearly 1 in 3 U.S. adults using them [2]. Their popularity is attributed to their efficacy in low-density lipoprotein cholesterol (LDL-C) reduction and CVD prevention [2]. However, despite nearly four decades of use and extensive research, the potential impact of statins on cognition and dementia remains a hotly debated topic [3]. At the same time, cognitive impairment and dementia pose a major public health burden and lack effective treatments [4].

The relationship between hypercholesterolemia and dementia is complex and not fully understood [5]. While midlife hypercholesterolemia is associated with a higher risk of cognitive decline and dementia, late-life hypercholesterolemia does not show the same risk, suggesting that cholesterol exposure duration may play a role [6]. However, life-long exposure to high cholesterol in those with familial hypercholesterolemia is not associated with an higher dementia risk [7]. Even so, the presence of atherosclerosis, which is closely related to hypercholesterolemia, has been associated with increased risk of dementia [8]. Given these findings, there is substantial interest in investigating whether lipid-lowering therapy with statins can help reduce the risk of cognitive impairment [9].

Since the release of the 2013 American College of Cardiology/American Heart Association (ACC/AHA) guidelines for the treatment of hypercholesterolemia, statin use has continued to rise [2]. These guidelines emphasize the use of high-intensity statins and dose intensification to optimize LDL-C reduction and prevent CVD. But as statin prescriptions have increased, so has interest in their potential effect on cognition [5]. In 2012, the U.S. Food and Drug Administration (FDA) issued a warning about potential cognitive impairment associated with statin use [10]. The data supporting this warning were mostly based on case reports and smaller observational studies [10]. Subsequent research has yielded mixed results, with studies reporting detrimental, neutral, or even protective effects of statins on cognition [11–18]. Notably, concerns about memory loss and cognitive changes are frequently reported as adverse effects of statins, which may discourage their use and potentially deprive patients of the cardiovascular benefits of statins [19]. It has been observed that the use of high-intensity statin therapy and dose intensification remains suboptimal among patients who would benefit from it [2]. Individuals for whom statin therapy is indicated but who do not adhere to it or discontinue treatment due to adverse effects, whether perceived or real, face an increased risk of CVD [20]. Concerns about harmful effect on cognition may undermine acceptance or adherence to statin therapy.

Data from several early studies raised concerns about an increased risk of cognitive impairment and dementia with statin use [12, 14], but more recent epidemiologic larger studies have not supported these findings [21–23]. Therefore, the purpose of this review is to examine the current evidence regarding the effects of statins on cognitive function, addressing ongoing debates and uncertainties in the field. While observational studies and smaller clinical trials have had inconsistent outcomes, many have been criticized for methodological issues, including potential biases such as confounding and reverse causation, as well as inconsistencies in the types of statins, dosages, and tools cognitive testing or methods used for the identification of dementia cases [3, 24].

Given the ongoing debate, the results of two well-designed randomized controlled trials (RCTs), STAREE (Study of STAtins for Reducing Events in the Elderly) and PREVENTABLE (Pragmatic evaluation of events and benefits of lipid lowering in older adults), are highly anticipated [25, 26]. These trials, with rigorous designs and comprehensive neurologic assessments, have the potential to provide a clearer understanding of the relationship between statin use and cognition, helping to resolve conflicting findings and guide future clinical recommendations.

### Statin Characteristics, Mechanisms and Cognition

Statins lower CVD risk through the reduction of LDL-C by inhibiting the HMGCR enzyme in the liver [27], but they also have anti-inflammatory and antioxidant effects [28]. It remains unclear whether statins have similar effects in the brain and how they might impact cognition [5]. Before examining the existing clinical evidence on how statins may impact cognition in clinical studies, it is important to first recognize the physiological differences between cholesterol homeostasis in the brain and peripheral tissues. The brain contains about 20% of the body’s cholesterol, most of which is metabolically dormant in myelin [29]. Brain cholesterol is synthesized locally by astrocytes, neurons, and oligodendrocytes and remains largely isolated from peripheral cholesterol due to the blood-brain barrier (BBB) [30, 31]. The BBB is impermeable to apolipoprotein B (apoB)-containing lipoproteins, including LDL-C, very low-density lipoproteins, and chylomicrons [31, 32]. Therefore, dietary cholesterol intake has a minimal effect on the level cholesterol in the brain. Furthermore, the amount of cholesterol in the human brain is determined in early development and remains relatively constant throughout adulthood [31]. Brain cholesterol is also metabolized differently compared to peripheral cholesterol. In the brain, a brain-specific pathway involving cholesterol 24-hydroxylase facilitates the metabolism and elimination of cholesterol from the brain [5]. Additionally, unlike peripheral cholesterol, which has a short half-life of hours to days, brain cholesterol is produced slowly and has a long half-life of up to 5 years [31]. As a result, statins can effectively lower peripheral cholesterol, without significantly affecting brain cholesterol levels [33]. Differences between brain and peripheral cholesterol are summarized in Table 1.

**Table 1:** Comparison of brain cholesterol and peripheral cholesterol

Characteristic	Brain cholesterol	Peripheral cholesterol
Distribution	• ≈ 25% of brain mass • Low uptake from circulation due to blood brain barrier (BBB) • About 80% contained in myelin sheaths	• < 1% of body mass • Peripheral cholesterol does not cross BBB • Located in multiple organs (muscle, liver, blood stream and adipose tissue)
Synthesis	• In situ synthesis in astrocytes and oligodendrocytes (< 2% of hepatic production) • Total amount determined early in life	• Mostly synthesized in the liver (≈ 80%) with high production rate • Amount variable throughout life by diet and other factors
Dietary Effect	• Levels minimally affected by dietary cholesterol intake	• Levels significantly affected by dietary cholesterol intake
Metabolism	• Hydroxylation to 24-hydroxycholesterol by 24-hydroxylase (a brain specific pathway) • Long half-life of months to years	• Hepatic conversion of cholesterol into bile acids via reverse cholesterol transport (RCT) • Short half-life of few days
Lipid-Lowering Agents	• Minimal effect	• Significant effect

Beside the physiological differences between peripheral and brain cholesterol, the potential effects of statins on cognition may also depend on specific characteristics of each statin. First, statins vary in their ability to cross the BBB according to their lipophilicity, possibly contributing to their differing effects on the brain [5]. More lipophilic statins like simvastatin, lovastatin, and atorvastatin are more likely to cross the BBB than hydrophilic statins like pravastatin and rosuvastatin [34]. However, current evidence is inconsistent regarding whether differences in the lipophilicity of statins influence their effect on cognition [15, 35, 36].

The timing, dosage and dosage-intensification, and duration of exposure to statin therapy have also been reported to influence its effects on cognition [35]. A study found that early statin use, defined as starting statins before acetylcholinesterase inhibitors, was associated with a reduced risk of disease progression in Alzheimer’s disease (AD) [37]. A duration–response relationship has been observed with one study finding a 9% reduction in dementia with each year of statin treatment [38]. Studies on the cognitive effects of statin dose intensification have produced conflicting results [35]. Some research suggests that higher doses of potent statins, like atorvastatin and rosuvastatin, may offer greater neuroprotection [39]. However, other studies have found that high-intensity statins (atorvastatin 40–80 mg, rosuvastatin 20–40 mg) have a stronger with cognitive impairment compared to moderate-intensity statins (atorvastatin 10–20 mg, rosuvastatin 10 mg, simvastatin 20–40 mg, and pravastatin 40–80 mg) [40]. The design of future clinical trials needs to include evaluating the impact of statin adherence on cognition, as most observational studies often lack this information.

Several patient factors may influence how statins affect cognition, including genetic factors such as LDL receptor (LDLR) status and apolipoprotein E (APOE) genetic variants [41]. Some evidence suggests that statins may reduce the risk of AD in individuals with the APOE ε4 allele [42]. Statins may also affect cognition through cholesterol-independent mechanisms such as amyloid-β (Aβ) cascade, tau phosphorylation, oxidative stress, apoptosis, neuroinflammation, and synaptic plasticity [43]. The aggregation of Aβ plaques and neurofibrillary tangles are key pathological mechanisms in AD, the leading cause of dementia [44]. There is a discrepancy in the literature regarding whether statins have an effect on the biomarkers of AD. A study found that statin therapy slowed Aβ deposition in cognitively healthy individuals but not in those with clinical signs of cognitive impairment [45]. However, another study showed that the effect of simvastatin on cerebral spinal fluid levels of phosphorylated tau181 in cognitively healthy individuals depends on baseline LDL-C levels [46].

**Fig. 1:**
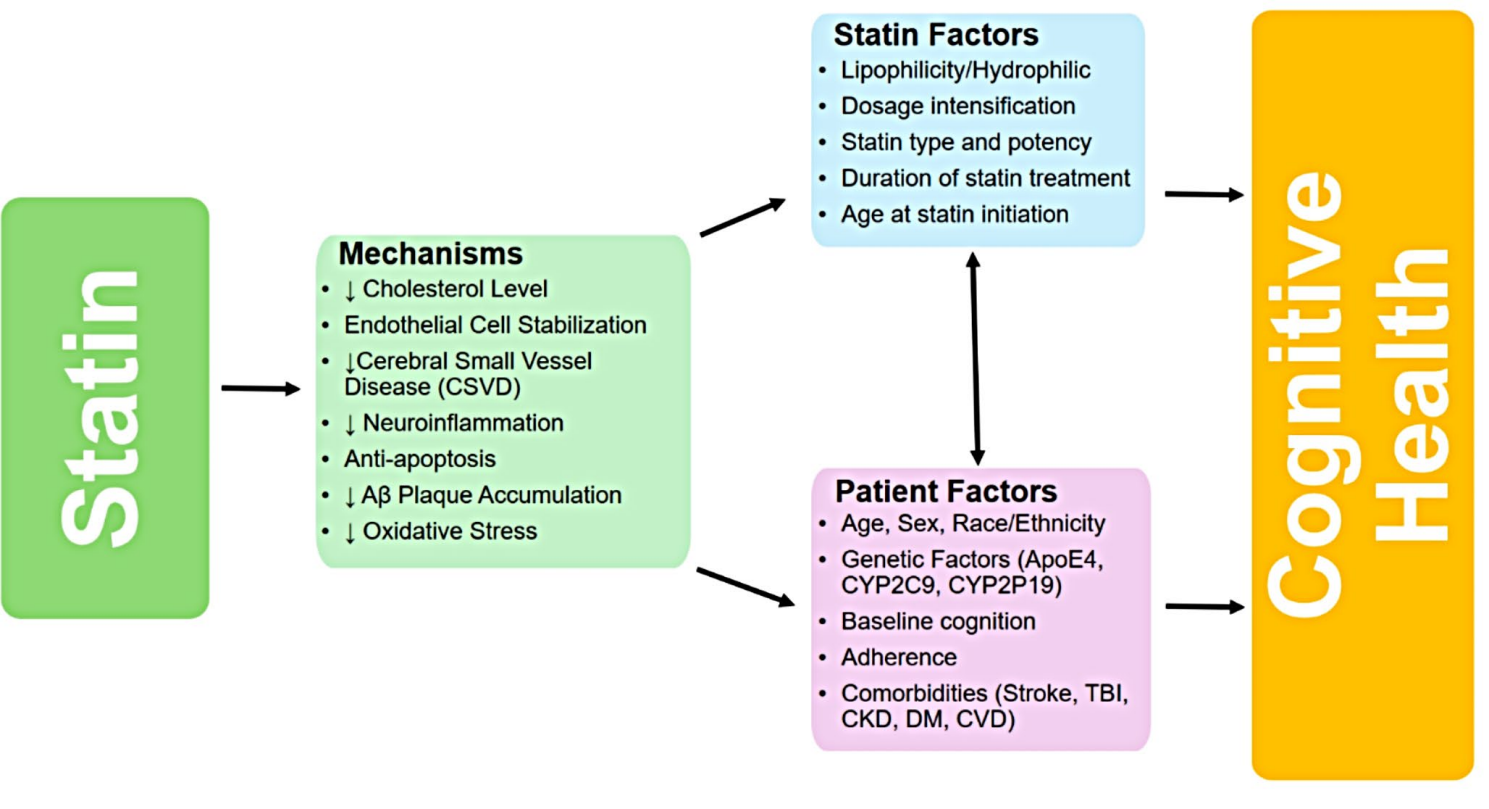
Potential mechanisms and modifying factors of statins’ effect on cognitive health. Abbreviations. Aβ, amyloid β; CNS, function. APOE4, apolipoprotein E isoform 4; CYP2C9, cytochrome P450 family 2 subfamily C member 9; CYP2P19, cytochrome P450 2 subfamily C member 19; CVD, cardiovascular disease; CKD, chronic kidney disease; DM, diabetes mellitus

Figure 1 illustrates the potential mechanisms and modifying factors influencing the effects of statins on cognition.

### Evidence for Negative Effects of Statins on Cognition

The FDA’s decision in 2012 to issue a warning about the potential negative effects of statins on cognition was based on small studies and case reports [10]. A 2009 patient survey of 171 individuals found that up to 75% of statin users reported cognitive-related adverse drug reactions (ADRs) [47]. In many of these studies, the cognitive effects were anecdotal, with varying onset times and inconsistent responses upon re-challenge [19]. Evidence from a few clinical trials available at the time was inconclusive [13, 14, 48, 49]. For example, a small clinical trial (*n* = 209) found that 20 mg of lovastatin was linked to reductions in attention and psychomotor speed [14]. In contrast, a separate trial (*n* = 283) using 10 mg and 40 mg of simvastatin reported only minor cognitive declines, which were likely influenced by baseline differences in the cognitive tests [13]. Both studies were not only limited by their small sample sizes but also by their short follow-up period of just six months, making it difficult to draw long-term conclusions.

Since 2012, a limited number of studies have suggested that statin therapy may be associated with cognitive impairment. For example, a large (*n* = 480,000) retrospective cohort study in 2015 reported a strong association between first-time statin use and the onset of acute, reversible memory loss occurring within 30 days of initial exposure (hazard ratio [HR] 4.40; 95% confidence interval [CI]: 3.01–6.41) [12]. However, a 2015 meta-analysis of 25 RCTs found no link between statin therapy and cognitive impairment, challenging the FDA’s warning [50]. More recent meta-analyses have generally shown either a neutral or even positive impact of statins on cognitive function [51–53]. Although only a handful of recent studies have linked statins to harmful cognitive effects, some evidence of these negative effects still persists. A recent Mendelian randomization study published in 2022 concluded that statins negatively impacted cognitive performance, reaction time, and cortical surface area, though they did not affect biomarkers of AD progression or the risk of Lewy body dementia [11].

### Evidence for Protective Effects of Statins on Cognition

Observational studies from the early 2000s suggested that statins might preserve cognitive function, though some reported little to no effect [54, 55]. In a United Kingdom case-control study, statins were associated with a more than 70% reduction in the relative risk of dementia [56]. Similarly, the Rotterdam Study linked both lipophilic and hydrophilic statins to a reduced risk of AD [57]. A nationwide survey in Taiwan found that statins, particularly the more potent types like atorvastatin and rosuvastatin, offered protective effects against dementia [39].

A meta-analysis found that each additional year of statin use reduced dementia risk by 20%, and every 5-mg increase in daily statin dosage lowered the risk by 11% [58]. Other studies have shown that long-term statin use is associated with a lower risk of AD, though short-term studies often report inconsistent effects [52, 59]. The follow-up duration appears to be crucial, as longer studies have demonstrated significant cognitive benefits compared to studies with shorter follow-up [59]. However, analysis of data from the Pravastatin in elderly individuals at risk of vascular disease (PROSPER) and the Heart Protection Study (HPS) studies failed to demonstrate any cognitive protective effects of pravastatin or simvastatin [48, 49]. The Cache County and Cardiovascular Health Studies found no association between statin use and reduced dementia risk [55, 60]. Meta-analyses of observational studies have also yielded mixed results, with some showing no significant effect of statins on dementia or AD [16, 61].

For the purposes of this review, relevant observational studies and clinical trials from PubMed, Google Scholar, Embase, and Scopus were identified, focusing on the last five years but including classic studies and extending the timeline where literature was sparse **(**Table 2**).**

**Table 2:** Summary of recent studies examining the impact of statins on cognitive function

Author (Year)	Design, size and follow-up	Statin and dose	Measure of cognition	Findings and conclusion	Limitations
**a. Summary of Studies Showing that Statins Have a Protective Effect on Cognition**					
Lee et al. (2020)[64]	• Prospective Cohort • *N* = 2386 • Follow-up = 12 years	Not defined	ICD codes	**Findings: Statin** use was associated with a reduced risk of dementia. For AD, the risk was also lower, while findings for VaD showed mixed results in both women and men. **Conclusions**: Among individuals with hypercholesterolemia, stain exposure was protective for all dementia and AD	Use of ICD codes for dementia. Statin type was not analyzed. Statin compliance not assessed.
Lee et al. (2020)[65]	• Retrospective cohort • *N* = 56,018 • Follow-up = 8 years	Not defined	ICD codes	**Findings**: Statin use was associated with a reduced risk of developing dementia. **Conclusion**: Periodontitis was a risk factor for dementia, while the use of statins may reduce the risk of dementia.	Use of ICD codes for dementia. Lack of information on socioeconomic status, lifestyle, laboratory data, and statin compliance not assessed.
Zingel et al. (2020)[17]	• Retrospective case-control study • *N* = 24,472 • Follow-up N/A	Multiple Statins	ICD codes	**Findings**: Statin use at all dosages was associated with a reduced risk of all-cause dementia and VaD. There was no significant association between LDL-C levels and dementia. **Conclusion**: Statin therapy at any dosage was negatively associated with all-cause dementia and VaD in elderly patients.	Severity of dementia not included Study did not include environmental factors known to affect cognition
Torrandell-Haro et al. (2020)[21]	• Retrospective cohort study • *N* = 288,515 • Follow-up = 5 years	Multiple statins	ICD-9 Codes	**Findings**: Statin use was associated with a lower incidence of AD and dementia. The benefit was greatest with pitavastatin and atorvastatin. **Conclusion**: Statins significantly mitigate neurodegenerative disease risk, including dementia	Study based on claims database. Dementia diagnosis based on ICD codes
Kim et al. (2019)[22]	• Retrospective Cohort • *N* = 143,174 • Follow-up = 5 years	Multiple statins	ICD codes	**Findings**: Among the 7 commonly prescribed statins, rosuvastatin showed the strongest preventive effect on dementia incidence, with some differences in effect based on sex. **Conclusion**: Appropriate statin use considering sex differences may have beneficial effects on the development of dementia.	used health insurance claims data severity of dementia was not included
Chang et al. (2019)[23]	• Longitudinal cohort study • *N* = 100,610 • Follow-up = 12 years	Multiple statins	ICD codes	**Findings**: Statin users had a lower risk of developing new-onset dementia (NOD) compared to non-users, with a stronger protective effect seen in those with higher statin exposure. **Conclusion**: Statin use is associated with a decreased NOD risk. The protective effect of statins for NOD seemed to be related to high exposure to statins.	ICD codes used for identifying dementia cases Retrospective design
Redelmeier et al. (2019)[37]	• population-based double cohort study • *N* = 28,815 • Follow-up = 3 years	Multiple statins	ICD codes	**Findings**: Patients with concussion who received statins had a 13% reduced risk of developing dementia compared to those who did not receive statins. Hydrophilic statins were marginally more beneficial than lipophilic statins. **Conclusion**: Statin use was associated with a reduced risk of dementia in older adults following a concussion.	Included only participants with concussion
Pan et al. (2018)[66]	• Cohort Study • *N* = 14,809 • Follow-up ≈ 8 years	Atorvastatin, fluvastatin, lovastatin, pravastatin, rosuvastatin, and simvastatin	ICD codes	**Findings**: Statin use was linked to a significantly lower incidence of dementia, with lipophilic and high-potency statins showing the greatest risk reduction. Longer statin exposure was associated with a further decreased risk of dementia. **Conclusion**: Statin use was associated with decreased risk of dementia among patients with stroke. The use of high-potency statins, lipophilic statins, and prolonged exposure to statins may be associated with greater benefits.	Use of ICD codes for dementia diagnosis Only included participants with stroke
b. **Summary of Studies Showing that Statins Have a Neutral Effect on Cognition**					
Zhou et al. (2021)[15]	• Secondary analysis of RCT (ASPREE) • *N* = 18,846 • Follow-up ≈ 5 years	Hydrophilic statins (Pravastatin and Rosuvastatin) Lipophilic statins (Atorvastatin, Simvastatin, Fluvastatin, Lovastatin and Pitavastatin)	Modified Mini-Mental State Examination (3MS) to measure global cognition SDMT, to measure psychomotor speed Hopkins Verbal Learning Test-Revised and delayed recall task to measure episodic memory Single letter (F) Controlled Oral Word Association Test to measure language and executive function	**Findings**: Statin use was not associated with a significant risk difference for MCI, MCI consistent with AD, other MCI, or incident dementia and its subtypes compared to non-use. **Conclusion**: In adults ≥65 years of age, statin therapy was not associated with incident dementia, mild cognitive impairment, or declines in individual cognition domains.	No data on prior use of statins, and no adjustment for APOE genotypes due to missing information, Limited generalizability.
Bosch et al. (2019)[16]	• Secondary analysis of RCT (HOPE-3) • *N* = 1626 • Follow-up ≈ 6 years	Rosuvastatin 10 mg	Digit Symbol Substitution Test (DSST) (primary outcome measure), the modified 12-item Montreal Cognitive Assessment (mMoCA), and the Trail Making Test Part B (TMT-B).	**Findings**: There was no significant difference in the odds of a ≥ 5-point decline in DSST scores between those taking rosuvastatin and placebo, or between those taking candesartan/hydrochlorothiazide plus rosuvastatin and double placebo. **Conclusion**: Rosuvastatin in combination with long-term blood pressure lowering, did not reduce the risk of cognitive decline.	• Incomplete data on cognitive function. • Selection bias by trial sites. • Participant self-selection
Chitnis et al. (2015)[67]	• Retrospective cohort • *N* = 8062 • Follow-up = 22 months	Not defined	ICD codes for dementia	**Findings**: Dementia rates were similar in current and former statin users. Inverse-probability-of-treatment weighting produced similar results. **Conclusion**: There was no difference in the risk of dementia among current and former users of statins as compared with nonusers in an already at-risk HF population.	• Statin types were not evaluated. • Generalizability was limited to HF patients.
Ancelin et al. (2012)[68]	• Prospective Cohort– The Three City Study • *N* = 6830 • Follow-up = 7 years	Unspecified lipophilic and hydrophilic statins	Global cognitive functioning, visual memory, verbal fluency, psychomotor speed, and executive function Clinical diagnosis of dementia	**Findings**: No significant associations were found between statins (irrespective of their lipophilicity) and either cognitive decline or dementia incidence. **Conclusion**: Statins were not associated with lower risk of dementia after adjusting for APOE and cholesteryl exchange transfer protein polymorphisms.	• Self-reported statin use
Trompet et al. (2010)[49]	• RCT– PROSPER • *N* = 5804 • Follow-up ≈ 4 years	Pravastatin 40 mg	Mini-mental status examination psychometric tests (picture-word learning test, Stroop color word test, letter digit coding test)	**Findings**: Pravastatin had no significant effect on cognitive function or disability. **Conclusion**: Pravastatin treatment in the elderly showed no impact on cognitive decline over three years, suggesting statins are ineffective for preventing cognitive decline in older adults.	• Trial was primarily designed for cardiovascular outcomes • Excluded those with cognitive function
Heart Protection Study (2002)[50]	• RCT • *N* = 20,536 • Follow-up = 5 years	Simvastatin 40 mg	Telephone Interview for Cognitive Status	**Findings**: Similar incidence rates of dementia in statin and non-statin groups **Conclusion**: Simvastatin use was not associated with a lower risk of cognitive impairment in this trial.	• Cognitive outcomes were secondary endpoint in the trial
c. **Summary of Studies Showing Statins Are Associated with Increased Risk of Cognitive Decline**					
Rosoff et al. (2022)[11]	• Mendelian Randomization • *N* = 740,000 • Follow-up N/A	Not applicable– potential neurocognitive impact of statin was proxied	Cognitive performance, reaction time, and cortical surface area Biomarkers of AD progression and LBD risk	**Findings**: HMGCR inhibition was linked to reduced cognitive performance, slower reaction time, and decreased cortical surface area. However, it showed no effect on biomarkers of AD progression or Lewy body dementia risk. **Conclusion**: Adverse neurocognitive effects from HMGCR inhibition were observed but may be outweighed by the cardiovascular benefits of statins.	• Study limited by assumptions of Mendelian Randomization design
Strom et al. (2015)[12]	• Retrospective Cohort • *N* = 482,542 • Follow-up = 30 days	atorvastatin, cerivastatin, fluvastatin, pravastatin, rosuvastatin, or simvastatin	Diagnostic codes with descriptions specifically pertaining to memory loss.	**Findings**: A strong association between first-time statin use and acute memory loss diagnosed within 30 days of initial exposure. **Conclusion**: Statins, and other lipid-lowering drugs, either cause acute memory loss or the association is the result of detection bias rather than a causal association.	• Short follow-up period • Intra-individual confounding, detection bias • Dosage of statins not assessed
Muldoon et al. (2004)[13]	• RCT • *N* = 283 • Follow-up = 6 months	Simvastatin 10 mg Simvastatin 40 mg	Neuropsychological test battery assessing cognitive functioning at baseline and at the end of the treatment period.	**Findings**: Simvastatin showed small negative effects on a few neuropsychological tests compared to placebo, with no improvement over 6 months, though baseline differences confounded one test. **Conclusion**: Partial support for minor decrements in cognitive functioning with statins, in the short-term, but long-term effects unknown	• Short follow up • Smaller sample size
Muldoon et al. (2000)[14]	• RCT • *N* = 209 • Follow-up = 6 months	Lovastatin 20 mg	Neuropsychological performance, depression, hostility, and quality of life were conducted at baseline and at the end of the treatment period.	**Findings**: Lovastatin-treated subjects showed improvement only in memory recall, while the placebo group had slightly greater improvements in attention and psychomotor speed. **Conclusion**: Lovastatin treatment for hypercholesterolemia did not cause psychological distress or significantly affect cognitive function, though minor declines in attention and psychomotor speed were observed.	• Small sample size • Short follow

Abbreviations AD, Alzheimer’s disease; VaD, vascular dementia; ICD, International classification of diseases; MCI, mild cognitive impairment; RCT, randomized controlled trial; ASPREE, ASPirin in Reducing Events in the Elderly; HOPE-3, Heart Outcomes Prevention Evaluation–3;PROSPER, Pravastatin in elderly individuals at risk of vascular disease; HMGCR, 3-hydroxy-3-methyl-glutaryl-coenzyme A reductase

### Statin Effects by Dementia Type

#### Alzheimer’s Disease (AD)

AD is the most common cause of dementia [66], primarily driven by the production and aggregation of Aβ and phosphorylated tau in the brain [44]. Aβ binds to cell membranes, leading to cell death in the brain [67]. Diet-induced hypercholesterolemia is linked to increased Aβ production and the progression of AD pathology [68]. Disturbances in brain lipid homeostasis can disrupt the blood-brain barrier, amyloid processing, endocytosis, myelination, signaling, energy metabolism, and increase inflammation. These lipid imbalances may contribute AD [69]. Furthermore, the APOE ϵ4 genotype, the most common genetic risk factor for AD, also plays a key role in lipid transport and metabolism [69]. Midlife hypercholesterolemia, compared to normal cholesterol levels, has been associated with a significantly higher risk of AD, with an estimated HR of 2.14 (95% CI 1.33–3.44) [6]. A retrospective study with nearly 12 years of follow-up found that statin use was linked to a reduced risk of AD, with HRs of 0.54 (95% CI: 0.32–0.91) for men and 0.53 (95% CI: 0.38–0.73) for women [18]. Similarly, a retrospective US-based Humana insurance claims study found that statin exposure was associated with a lower incidence of AD (RR 0.46; 95% CI, 0.44–0.49) [21]. A pooled analysis from a recent meta-analysis of 21 studies found that statin use was associated with a 32% lower risk of AD (odd ratio [OR] 0.68, 95% CI 0.56–0.81). This analysis also showed that both lipophilic and hydrophilic statins provided cognitive benefits. Moreover, high-potency statins were linked to a 20% reduction in AD risk, compared to a 16% reduction with low-potency statins [51]. The mechanisms linking statins to AD are not fully understood, but some studies suggest they may exert beneficial effects by slowing Aβ aggregation [45]. Nonetheless, the protective effect of statins on AD risk seems to be independent of their lipophilicity, although more studies are needed to confirm this [57]. In contrast, other studies have found no significant association between statin exposure, irrespective of their lipophilicity, and the risk of AD [11, 65]. The majority of the extant evidence suggests statins are associated with either a reduced risk of AD or no significant impact. However, much of this evidence comes from observational studies, which largely report protective effects [51]. Therefore, ad hoc randomized clinical trials are needed to confirm these findings.

#### Vascular Dementia

Vascular dementia (VaD) is the second most common type of dementia after AD, accounting for around 15% of dementia cases [70]. VaD is primarily caused by cerebrovascular disease and vascular risk factors; thus, hypercholesterolemia is considered a potential risk factor for VaD [71]. Although midlife hypercholesterolemia is associated with an increased risk of VaD [6, 72], late-life hypercholesterolemia, on the contrary, may have a protective effect [73]. This suggests cholesterol levels’ effect on cognitive health might vary depending on the stage of life. Hypercholesterolemia and low-grade inflammation contribute to endothelial dysfunction, reducing blood flow (chronic cerebral hypoperfusion) and disrupting the BBB. Dyslipidemia further exacerbates hypoperfusion, promoting brain inflammation and cognitive decline seen in VaD [71]. Due to the close relationship between cerebral vascular disease and VaD, this type of dementia is theoretically the most likely to benefit from statin therapy [74]. Despite this, there are limited studies investigating the potential effect of statin on VaD. In the study of individuals with diabetes, statin use was associated with a reduced risk of VaD, with an OR of 0.86 (95% CI: 0.81–0.91) [75]. Similarly, a cross-sectional study reported a significantly lower risk of VaD in statin users compared to non-users, with an OR of 0.25 (95% CI: 0.08–0.85) [76]. Other studies have shown that while statin therapy reduced the risk of all-type dementia, AD, and mild cognitive impairment, it had no effect on incident VaD [54, 77]. Several factors have been identified as potential reasons for these inconsistencies, including short follow-up periods, variations in statin types, inclusion of patients with advanced VaD, differences in cognitive testing methods, and the possibility of distinct pathophysiological mechanisms in VaD. These issues underscore the need for well-designed clinical trials to clarify the impact of statins on VaD [78].

#### Lewy Body Dementia (LBD)

Lewy body dementia comprises of both dementia with Lewy bodies and Parkinson’s disease dementia [79]. Lewy bodies are abnormal aggregations of the α-synuclein proteins in neurons. LBD is the third leading cause of dementia and has no curative treatment [66]. Patients with LBD disproportionately experience greater impairments in attention, executive function, and visuoperceptual abilities compared to impairment in memory. However, LBD shares considerable overlap in both its histopathology and clinical symptoms with AD and VaD [79]. There is a scarcity of studies exploring the relationship between serum lipids and the risk of LBD. Although some studies have associated elevated serum LDL-C and low levels of high-density lipoprotein cholesterol (HDL-C) with an increased risk of LBD, it remains unclear whether statins have any impact on modifying this risk [80, 81]. A study of dementia patients with autopsy-confirmed Lewy bodies found a lower prevalence of CVD and its risk factors in LBD compared to VaD, raising questions about the influence of CVD risk factors such as hypercholesterolemia on LBD risk [82]. But a large case-control study investigating the effects of various cardiovascular drugs found that cholesterol-lowering agents were linked to a reduced risk of LBD, with an odds ratio of 0.85 (95% CI 0.83–0.87) [83]. That said, most epidemiological studies on statins’ cognitive effects have not addressed LBD as a subtype of dementia. Future clinical trials should prioritize filling this research gap.

#### Frontotemporal Dementia (FTD)

FTD is characterized by behavioral changes or language problems. Changes in affect, along with a lack of concern and insight, are key factors that distinguish FTD from AD and VaD [84]. FTD is considered to be the fourth leading cause of dementia across all age groups [66]. FTD is associated with neuro-apoptosis affecting the front and temporal lobes of the brain leading to atrophy [85]. The extant literature on the role of lipids and lipid-lowering therapies in the development of FTD is notably sparse. Data based on Mendelian randomization found that among different lipids, only apolipoprotein B (ApoB) level was associated with FTD risk [86]. In the same study, authors reported that using genetic proxies for drug targets, there was a potential effect of targeting APOB as a means to reduce the LDL-C levels and the risk of FTD OR 0.58(95% CI 0.39–0.87). This observation suggests a potential protective role of HMGCR inhibition with statins [86]. But this limited research leaves significant gaps in understanding the potential relationship between lipid metabolism, statin therapy and FTD, highlighting the need for further investigation in this area.

### Mild Cognitive Impairment (MCI)

MCI is an intermediate state between age-related cognitive decline and dementia [87]. It is estimated that over 15% of adults aged 50 and older are affected by MCI [88], with 20–40% of these individuals likely to progress to dementia [89]. Similar to dementia, midlife hypercholesterolemia is a significant risk factor for MCI (OR = 1.9, 95% CI: 1.2–3.0) [90]. Vascular risk factors, including hypertension, diabetes, and hypercholesterolemia, are associated with an increased risk of MCI progressing to dementia [91]. An analysis of the ADNI study indicated that while statin therapy did not affect the progression of late MCI, it showed some protective effects in individuals with early MCI [92]. In a pooled analysis of six studies, statin use was linked to a reduced risk of incident MCI (adjusted RR = 0.737, 95% CI: 0.56–0.98) [77]. Future clinical trials are needed to explore how statins may influence the onset of MCI and its progression.

### Impact on Cognition in Selected Populations

#### Older Individuals

Age is a major risk factor for both cognitive impairment and CVD, underscoring the importance of statin therapy in older individuals. However, statin discontinuation rates are high in older adults, which is associated with an increased risk of CVD events [20]. Studies have shown inconsistent associations between statin use and cognitive function in older individuals. Some have found a protective effect against dementia, particularly in those with the APOE ε4 allele, while others have reported no significant association [17, 42]. Systematic analyses have generally concluded that statin therapy does not significantly increase dementia or AD risk in older adults, but existing trials have significant limitations, such as small sample sizes and short follow-up periods [93, 94].

#### Prevalent Dementia

In patients with existing dementia, the role of statins in slowing disease progression is uncertain. While some studies have shown that statins may lower all-cause mortality in dementia patients, guidelines are unclear about their use for CVD prevention in this population [95, 96]. Some observational studies have suggested a dose-response relationship between statin use and cognitive improvement, but findings are inconsistent [97]. Contemporary clinical trials investigating the effects of statins in patients with AD are needed, as few have been conducted in the past decade. However, a 2020 meta-analysis of clinical trials revealed that while statin use in AD patients had short-term beneficial effects on Mini-Mental State Examination (MMSE) scores, it did not result in improvements in AD Scale-Cognitive Subscale (ADAS-Cog) scores [98].

#### Stroke

Stroke patients have a higher risk of developing dementia compared to those without stroke [99], but the cognitive benefits or harms of statins in these patients are unknown. An analysis of data from Taiwan’s national database of stroke patients found that statin therapy was associated with a reduced risk of developing dementia (HR 0.81, 95% CI: 0.73–0.89). The protective effect was more pronounced with lipophilic statins and increased with prolonged exposure [63]. In another study, initiating statins after an ischemic stroke significantly reduced the risk of dementia (HR 0.70, 95% CI: 0.64–0.75), with potentially greater benefits observed in patients who continued statin therapy over time [100]. A systematic review found that post-stroke statin use was associated with a reduced risk of cognitive impairment but did not significantly protect against dementia [100]. In contrast, the Prevention of Decline in Cognition after Stroke Trial (PODCAST) trial found that, following an ischemic stroke, intensive blood pressure control and lipid-lowering were safe and feasible. Additionally, intensive lipid-lowering was associated with an improvement in the Addenbrooke’s Cognitive Examination-Revised score [101]. However, other studies have found a higher risk of hemorrhagic stroke with the initiation of high-intensity statins in patients with recent ischemic stroke [49, 102].

### Effect of Sex and Race/Ethnicity

Nearly two-thirds of all dementia cases occur in women, and older African Americans are almost twice as likely to develop dementia compared to older white individuals [4]. In a study of nearly 400,000 Medicare participants, simvastatin and atorvastatin were broadly protective against AD, while pravastatin and rosuvastatin reduced AD risk only in white women. However, high statin exposure did not lower AD risk in black men [103]. Another study showed that different statins have differential effects on cognition based on sex [22]. In two meta-analyses of observational studies, the risk of dementia was decreased both in male and female patients with statins [51]. Future studies are needed to confirm any differential effect of statin by sex and race/ethnicity.

### Review of Current Guidelines

There is limited evidence on safety and efficacy of statins in older adults with dementia, particularly when used for CVD prevention [96]. The existing major guidelines on CVD prevention with statins have not universally addressed this issue [96]. Both the 2013 ACC/AHA and 2016 US Preventive Services Task Force (USPSTF) guidelines concluded that there was no evidence indicating that statins had an adverse effect on cognitive changes or the risk of dementia. However, they did not address whether statins might offer any protective benefits [1, 104]. The USPSTF acknowledged that there is a scarcity of data to assess the benefits and harms of starting statins in individuals aged 76 years or older [104]. However, the 2019 ACC/AHA guideline on the primary CVD prevention did not address the potential effect of statins on cognition [105]. A similar trend of limited information and recommendations regarding statins and cognitive impairment is observed in other major current international guidelines, highlighting the need for future clinical trials to provide concrete data [96].

## Conclusion

Cognitive impairment and dementia pose a major public health burden. Statins are highly effective in treating hypercholesterolemia and preventing CVD, but there are unresolved controversies about their effect on cognitive health. Since cholesterol homeostasis in the brain differs from that in peripheral tissues, the effects of statins on circulating cholesterol may not necessarily extend to the brain and its function.

Although some might reasonably argue, based observational data, that statins provide a neurocognitive benefit, most would agree that harmful effects of statins on cognition are much less evident in the existing literature. That said, concerns about the cognitive side effects of statins may discourage some patients from continuing or adhering to statin therapy, which is important for CVD prevention. The current evidence on the link between statins and cognitive health is limited, as it largely comes from observational studies with methodological weaknesses, including bias, confounding, and reverse causation. Additionally, inconsistent findings across studies make it difficult to draw definitive conclusions about the cognitive risks or benefits of statins. Another characteristic of current studies is that short-term research often reports no effect or adverse cognitive outcomes, while long-term studies tend to suggest cognitive benefits. To resolve these uncertainties, well-designed RCT trials are needed to test whether statins protect against or contribute to cognitive decline. The ongoing PREVENTABLE and STAREE trials are expected to provide valuable insights into statins’ effects on cognition and dementia-related disability in older individuals. However, these trials may not fully address the effects of statins in specific populations, such as those with diabetes, CVD, chronic kidney disease, concussion and traumatic brain injury, or hypertension. Future studies will still be needed to investigate the impact of statins in these high-risk groups.

## Key References

- Goldstein LB, Toth PP, Dearborn-Tomazos JL, et al: Aggressive LDL-C lowering and the brain: impact on risk for dementia and hemorrhagic stroke: a scientific statement from the American Heart Association. Arteriosclerosis, Thrombosis, and Vascular Biology 2023, 43(10):e404-e442. 10.1161/ATV.0000000000000164.
  A contemporary statement from the American Heart Association (AHA) that critically evaluates evidence on the relationship between LDL-C reduction and its impact on cognitive impairment and dementia. It also examines whether more intensive statin therapy offers additional cognitive benefits or risks. The comprehensive analysis provided makes this a key source for understanding the complex link between lipid management and cognitive health, offering essential insights for ongoing research.
- Olmastroni E, Molari G, De Beni N, et al: Statin use and risk of dementia or Alzheimer's disease: a systematic review and meta-analysis of observational studies. Eur J Prev Cardiol 2022, 29(5):804-814. 10.1093/eurjpc/zwab208.
  A large, recent systematic review and meta-analysis that compiles evidence from observational studies, which constitute the majority of existing research, on the potential effects of statins on cognitive decline and dementia.
- Matyori A, Brown CP, Ali A, Sherbeny F: Statins utilization trends and expenditures in the U.S. before and after the implementation of the 2013 American College of Cardiology/American Heart Association (ACC/AHA) guidelines. Saudi Pharm J 2023, 31(6):795-800. 10.1016/j.jsps.2023.04.002.
  The reference explores statin use in the United States before and after the 2013 ACC/AHA cholesterol treatment guidelines. It highlights a key challenge: suboptimal prescribing of high-intensity statins, possibly driven by concerns about their potential cognitive side effects among patients.

## Data Availability

No datasets were generated or analysed during the current study.
